# Evaluation of a multimedia youth anti-smoking and girls’ empowerment campaign: SKY Girls Ghana

**DOI:** 10.1186/s12889-020-09837-5

**Published:** 2020-11-17

**Authors:** Paul Hutchinson, Alejandra Leyton, Dominique Meekers, Charles Stoecker, Francine Wood, Joanna Murray, Naa Dodua Dodoo, Adriana Biney

**Affiliations:** 1 Tulane University School of Public Health and Tropical Medicine, New Orleans, LA USA; 2 Development Media International, London, UK; 3 University of Ghana, Accra, Ghana

**Keywords:** Smoking prevention, Impact evaluation, Multimedia intervention

## Abstract

**Background:**

Given the long-term health effects of smoking during adolescence and the substantial role that tobacco-related morbidity and mortality play in the global burden of disease, there is a worldwide need to design and implement effective youth-focused smoking prevention interventions. While smoking prevention interventions that focus on both social competence and social influence have been successful in preventing smoking uptake among adolescents in developed countries, their effectiveness in developing countries has not yet been clearly demonstrated. SKY Girls is a multimedia, empowerment and anti-smoking program aimed at 13–16-year old girls in Accra, Ghana. The program uses school and community-based events, a magazine, movies, a radio program, social media and other promotional activities to stimulate normative and behavioral change.

**Methods:**

This study uses pre/post longitudinal data on 2625 girls collected from an interviewer-administered questionnaire. A quasi-experimental matched design was used, incorporating comparison cities with limited or no exposure to SKY Girls (Teshie, Kumasi and Sunyani). Fixed-effects modeling with inverse probability weighting was used to obtain doubly robust estimators and measure the causal influence of SKY Girls on a set of 15 outcome indicators.

**Results:**

Results indicate that living and studying in the intervention city was associated with an 11.4 percentage point (pp) (95% CI [2.1, 20.7]) increase in the proportion of girls perceiving support outside their families; an 11.7 pp. decrease (95% CI [− 20.8, − 2.6]) in girls’ perception of pressure to smoke cigarettes; a 12.3 pp. increase (95% CI [2.1, 20.7]) in the proportion of girls who had conversations with friends about smoking; an 11.7 pp. increase (95% CI [3.8, 20.8]) in their perceived ability to make choices about what they like and do not like, and 20.3 pp. (95% CI [− 28.4, − 12.2]) and 12.1 pp. (95% CI [− 20.7, − 3.5]) reductions in the proportion agreeing with the idea that peers can justify smoking shisha and cigarettes, respectively. An analysis of the dose-effect associations between exposure to multiple campaign components and desired outcomes was included and discussed.

**Conclusion:**

The study demonstrates the effectiveness of a multimedia campaign to increase perceived support, empowerment and improve decision-making among adolescent girls in a developing country.

**Supplementary Information:**

The online version contains supplementary material available at 10.1186/s12889-020-09837-5.

## Introduction

### Background

Given the long-term health effects of smoking during adolescence, there is a need to design and implement effective and culturally relevant youth-focused smoking prevention interventions worldwide. Smoking prevention interventions that focus on both social competence and social influence have been successful in reducing/delaying smoking uptake among adolescents in developed countries [5, 28]. However, it is unclear whether this approach can be equally effective in developing country settings. This paper measures the impact of SKY Girls Ghana, a youth-focused smoking prevention intervention that uses multiple communication channels to empower girls and to enhance their ability to make healthier decisions. The results of the study have important implications for the potential scale-up of the SKY Girls campaign, including its possible replication in other settings, as well as for social empowerment and social influence interventions targeting other adolescent health behaviors.

Tobacco use, specifically smoking, is the leading cause of death worldwide, with 6 million tobacco-related deaths every year [31]. Globally, nearly 90% of daily cigarette smokers first try cigarettes before the age of eighteen [29, 32], with early onset of smoking being associated with greater risk of negative health outcomes and premature death [11, 19]. In Ghana, recent data show that smoking prevalence is low among 13–15-year-olds attending school (8.7% ever smoked cigarettes and 8.8% ever smoked shisha) [17]. However, repeated cross-sectional data reveal an important increase in the proportion of 13–15-year-old non-smokers who believe they might smoke in the next 12 months if offered by a friend (from 14.6% in 2005 and 15.9% in 2009 to 21.6% in 2017) [15–17].

Previous research demonstrates that smoking during adolescence is a multi-determined behavior influenced by numerous interpersonal factors, social norms and social networks [4, 22, 24, 29, 34]. During this life stage, adolescents typically explore their own individual identities and seek group membership in their own social environment, making them highly susceptible to peer influence on risk taking. If smoking is deemed to be “cool” among peers, an environment can emerge/exist in which adolescents feel pressure to adopt smoking behaviors to achieve social acceptance [4, 8, 24, 30].

Given the multi-determined nature of smoking behaviors, interventions that prevent smoking initiation have usually focused on (i) delivering information to oppose tobacco use [27]; (ii) improving broader life and social skills (i.e., social competence) to help adolescents refuse offers to smoke [28]; (iii) increasing awareness of the social influences that support smoking and improving abilities to deal with peer pressure [6, 25]; and (iv) a combination of social competence and social influences strategies [12, 13, 23]. By focusing on individual decision-making ability, interventions that combine social competence and social influences strategies can help empower adolescents and improve their capacity to make decisions that are consistent with their intentions, despite perceived social pressure.

Because there is scant evidence of the effectiveness of smoking prevention programs that combine social competence and social influences strategies in developing countries, this study aims to address that evidence gap by measuring the impact of the SKY Girls intervention in Ghana.

### The SKY Girls Ghana campaign

**Fig. 1:**
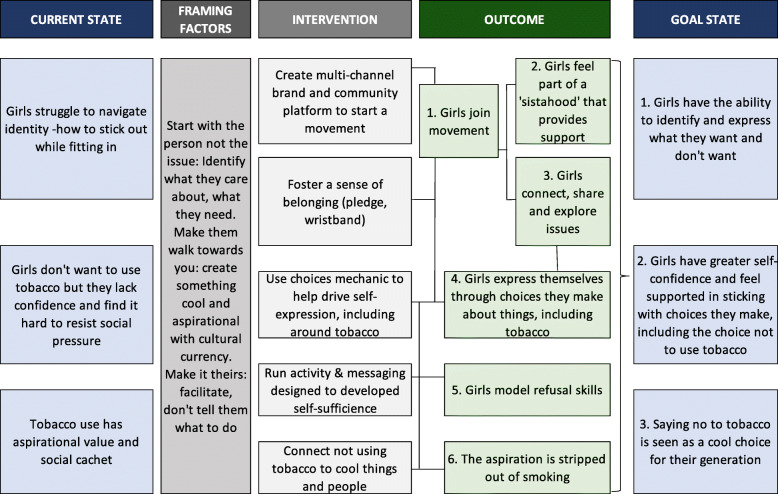
Theory of Change (TOC) for the SKY Girls Ghana campaign. Source: Good Business, personal communication

Designed and implemented by Good Business (GB) and Now Available Africa (NAA) following similar interventions in Uganda and Botswana, SKY Girls Ghana uses the tagline “Be true to yourself” to build an aspirational environment in which adolescents feel a sense of social identity and social inclusion. According to the program’s Theory of Change (TOC) (Fig. 1), this aspirational environment encourages girls to act in ways that are beneficial to their health and well-being. Furthermore, by stripping out the aspirational aspects of smoking, SKY Girls seeks to reduce positive attitudes associated with tobacco use and intentions to use, while increasing the ability to resist social pressure among young people at risk of using tobacco.

As a multimedia campaign, SKY Girls relies on 12 components to stimulate normative and behavioral change. As an entry point for their target audience, SKY uses school visits or “activations” with junior and senior high schools in Accra. During school visits, SKY Girls facilitators introduce girls to the principles of the “SKY sistahood,” and encourage girls to take the SKY pledge, a commitment device for girls to remain true to themselves and to say no to tobacco. In addition, other major campaign components consist of airing a weekly radio program; composition of a SKY Girls song; creation of social media handles on Instagram, Facebook and YouTube; distribution of magazines in print and online; and, production of two movies shown in movie theatres across Accra and available on the SKY Girls’ YouTube Channel. SKY Girls also organizes events with local celebrities at shopping malls in Accra. On weekends, a SKY truck drives through local communities to invite girls to SKY pop-up areas where they can take part in SKY activities. A detailed description of SKY Girls main components and activities is presented in Table 1.

**Table 1:** SKY Girls Ghana components

Component		Description
1	Magazine	Distribution of magazines in print and online. Two issues were released in 2017 and five in 2018. Key objectives for the magazines were to inspire SKY Girls with smoke-free role models, to be future-oriented in their decisions and to reject shisha and cigarettes if offered, all couched within aspirational content and passion points for teen girls, such as music and fashion. (http://www.skygirlsgh.com/magazine.html)
2	School activations/ visits	Implementation of school visits. During school visits, SKY Girls introduced girls to the principles of the “SKY sistahood,” deepened understanding of core messages (i.e., “Be true to yourself”), encouraged girls to express themselves using choice mechanics for what they like and do not like, and promoted all SKY events.
3	School clubs	The SKY Girls school clubs were launched in June 2018 as school-based events during lunch hours or after classes. Girls in the clubs were encouraged to take the SKY pledge (a commitment device), and each week covered different topics related to SKY Girls outcomes.
4	Parties at the mall	Organization of SKY Girls parties at shopping malls in Accra. These had the objectives of bringing girls together and building a sense of community. These were aspirational events with different activities (e.g., photo booth, career booth, sign up booth) and influencers on stage to help spread the campaign’s message.
5	Radio show	Weekly broadcast of the SKY Girls radio show on youth station YFM. Presented by three SKY Girls and a radio host, the girls covered a mix of topics from fashion hacks to how to deal with toxic friendships.
6	Community activations/ community truck	On weekends, a SKY truck would drive through local communities and invite girls to pop-up areas where girls could take part in activities. These events started in August 2018 and were also used to help distribute the seventh issue of the SKY Girls magazine.
7	Social media	Social media activities were conducted through channels such as Facebook, Instagram and YouTube. Posts shared on social media included advice related to questions asked by girls, thematic posts for the month, promotional posts for events/other activities, trivias and photos from school activations or events. https://www.facebook.com/skygirlsgh/ https://www.youtube.com/channel/UCp_ob3_d0UCmvIBBJteDAEA
8	Movie	Production of two SKY Girls movies. The movies aired on television in May and September of 2018, were shown in movie theatres across Accra, and were widely shown in school “movie night” activations. Both movies are also available on the SKY Girls’ YouTube Channel. Key objectives of the movies included building understanding of core intervention messages (e.g., “Be true to yourself”) and stripping the aspiration out of smoking. Sugar, Spice and Sauce: https://www.youtube.com/watch?v=dsl4egtMnig Sugar, Spice and Sauce 2: https://www.youtube.com/watch?v=9irvnuHVj2A
9	Song	Production and dissemination of SKY Girls Songs. “Unstoppable” is the first SKY Song broadcasted on local radio and YouTube. The song is performed by Cina Soul and the lyrics promote confidence and reinforce the “Be true to yourself” message. https://www.youtube.com/watch?v=1-Icy6NViH8
10	SMS messaging	Starting in November 2017, weekly messages were sent to SKY Girls who owned their own mobile phone. Messages included inspirational quotes, details about upcoming events and core intervention messaging.
11	WhatsApp broadcasting	Using WhatsApp as a platform, groups with several girls were created for the promotion of SKY Girl events, movies and the radio show SKY Live.
12	Vlog	Facebook and YouTube vlogs started in June 2018, providing aspirational content to inspire girls with smoke-free role models.

## Methods

To estimate the impact of SKY Girls Ghana, we used two approaches: 1) an intent-to-treat analysis at the geographic level, and 2) a dose-response analysis at the individual level. Specifically, we first use a quasi-experimental design and longitudinal data from pre- and post-surveys among a representative sample of girls living and/or studying in the intervention city (Accra) and in three comparison cities (Teshie, Kumasi and Sunyani) to test the overall effect of the intervention. Because SKY Girls is a multi-component program, we subsequently use dose-response analyses to measure the effect of different levels of exposure to the campaign.

### Data and population

Data were collected from two face-to-face surveys, a baseline survey in early 2017 (just prior to the initiation of SKY Girls activities), and an endline survey in late 2018 (as the program was nearing completion). Data collection was conducted by the Office of Research, Innovation and Development (ORID) of the University of Ghana, with technical assistance from the Tulane School of Public Health and Tropical Medicine.

The target population for the 2017 baseline survey consisted of youth aged 13–16 living in the intervention city (Accra) and the three comparison cities (Teshie, Kumasi, and Sunyani), with a total target sample size of 7200 (1800 per city including 900 boys and 900 girls). The sample was drawn using a multi-stage sampling strategy. Using data from the 2010 Ghana Population and Housing Census [14], 281 enumeration areas (EAs) were drawn as follows. In Accra, the sample was purposively restricted to ten localities within the catchment area of the SKY Girls intervention (Achimota, Spintex, Legon, Madina, Adenta, Dome, St. Johns, Christian Village, Haatso, and Kisseman). Consequently, the Accra sample is not representative of all of Accra. Within the localities in the SKY Girls catchment area, 70 out of a total of 380 census enumeration areas (EA) were drawn using probability of selection proportional to size (PPS). In Kumasi, the Ghana Statistical Service first restricted the sample to 16 localities matched to the Accra localities based on their social and economic characteristics (Nhyiaeso, Asuoyeboa, Aboabo, Asokwa, Old Tafo, Kwadaso, Ash Town, Bantama, Old Suame, Dichemso/Yenyawoso, Ayigya, Asafo, Atonsu and New Suame). Within these matched localities, 79 out of 733 EAs were selected with PPS. In Teshie and Sunyani, EAs were selected through simple random sampling (50 out of 220, and 78 out of 101, respectively). Within the selected EAs, all households were contacted and a household listing was conducted to determine whether the household was eligible for inclusion in the study. All eligible households were visited, and up to three attempts were made to interview all eligible household members aged 13–16.

Fieldwork for the baseline survey was implemented between March 28 and May 11, 2017. The overall response rate for the baseline survey was 91.7% (ranging from 87.2% in Teshie to 96.0% in Sunyani), which yielded a total sample size of 7054 respondents. The endline survey aimed to re-interview these same respondents. The initial phase of the fieldwork was conducted from November 13 through December 3, 2018. To address difficulties contacting and interviewing youth who were in boarding school, the fieldwork period was extended through December 26, 2018. To minimize the dropout rate across the two surveys, some youth who could not be reached for face-to-face interviews were re-interviewed by phone (provided that they had previously consented to be re-contacted by phone). In total, 5147 youth were successfully re-interviewed (of whom 999 were interviewed by phone), yielding a retention rate of 73.0% across survey waves. Our analysis is limited to female respondents, which reduced our working sample to 3775 girls at baseline and 2625 at endline.

The baseline questionnaire enquired about the respondent’s socioeconomic background, tobacco knowledge, exposure to messages about tobacco, beliefs and attitudes regarding shisha and cigarette smoking, empowerment, social norms and social influences with respect to smoking, as well as smoking behaviors and intentions. The endline questionnaire was nearly identical, with questions added about exposure to the SKY Girls program. The survey questionnaires were designed specifically for this study [18]. Some questions however were modeled after those in the Global Youth Tobacco Surveys [33].

### Ethical approval

Ethical clearance for the data collection was provided by the Institutional Review Board of the Noguchi Memorial Institute for Medical Research in Ghana (#078/16–17), and subsequently renewed in January 22, 2018. The study protocol was also reviewed and approved by the Tulane University Biomedical Institutional Review Board. Parental consent and child assent were obtained prior to the start of the interviews [9, 10].

### Measures

#### Outcome measures

The analysis focuses on 15 indicators intended to measure the expected program outcomes. Outcome measures are related to concepts of perceived social support, empowerment, decision-making agency as well as beliefs and social norms about smoking shisha and/or cigarettes. Detailed information about the definition and measurement of the 15 outcome indicators used in this analysis is presented in Table 2.

**Table 2:** Definition and operationalization of outcome indicators

Outcome	Outcome indicator (expected direction of the association in parenthesis)	Description
1. Girls feel part of a ‘sistahood’ that provides support	1. Perception of sources of social support outside the family (+) 2. Perception that friends support their decisions (+) 3. Perception of acceptance when saying no to friends (+) 4. Perception of pressure to smoke shisha (−) 5. Perception of pressure to smoke cigarettes (−)	% who agree or strongly agree with the statements: *1. “I have sources of support outside my family”* *2. “My friends support all my decisions, even if they don’t agree with them”* *3. “My friends think I’m cool if I say no to things I don’t want to do even when everyone else is doing it”* % who agree or strongly agree with the statement: *4. “People my age feel pressure to smoke shisha”* *5. “People my age feel pressure to smoke cigarettes”*
2. Girls connect, share and explore issues	6. Held conversations with friends about smoking (+) 7. Held conversations with adults about smoking (+)	6.% having any conversations in the last 2 months with friends about smoking 7.% having any conversations in the last 2 months with adults about smoking
3. Girls express themselves through choices they make about things, including tobacco	8. Perception of being able to make choices for themselves (+)	% who agree or strongly agree with the statement: *8. “I feel able to make choices about what I like and do not like for myself”*
4. Girls model refusal skills	9. Perception of confidence when saying no to friends (+)	% who agree or strongly agree with the statement *9. “I’m confident I can say no to things I do not want to do, even when everyone else is doing it*
5. The aspiration is stripped out of smoking	10. Agreement with beliefs that favor smoking shisha (−) 11. Agreement with beliefs that favor smoking cigarettes (−)	10 & 11. % having a high score (versus low) in the summation of six binary variables representing agreement or strong agreement with five positive attributes of smokers (smoking is cool; boys who smoke are attractive; girls who smoke are attractive; youth who smoke are popular; youth who smoke are grown-up) and one positive attribute of non-smokers (non-smokers are cool (variable was reversed coded in the index))
	12. Agreement with the idea that peers can justify smoking shisha (−) 13. Agreement with the idea that peers can justify smoking cigarettes (−)	12 & 13. % having a high score (versus low) in the summation of seven binary variables representing agreement or strong agreement with justifications a friend would use to smoke (to fit in with a group, to be popular with girls, to be popular with boys, to look rebellious, to look grown up, to stand out, to show they can make decisions for themselves).
	14. Perception that most peers smoke tobacco (−) 15. Perception that most peers smoke shisha (−)	% who agree or strongly agree with the statement: *14. “Most people my age smoke tobacco”* *15. “Most people my age smoke shisha”*

#### Measures of exposure to SKY Girls

##### Intent-to-treat analysis at the geographic level

The city of Accra was selected as the target location for the implementation of SKY Girls. Hence, all community-based, school-based and mass media-based activities targeted girls living in selected neighborhoods of the city. However, baseline results evidenced internal migration between cities in Ghana, and some girls lived in one city but studied in a different one (e.g., girls attending boarding schools). Therefore, and for the purpose of this evaluation, we classify respondents into three potential treatment groups: (i) full treatment, (ii) partial treatment, and (iii) no treatment. The full treatment group includes girls who both lived and attended school in the target location (i.e., Accra) at the time of the baseline survey. The partial treatment group includes girls who either lived in Accra or went to school there, but not both. The remaining girls are classified as “no treatment.” These are mostly girls living and studying in Kumasi and Sunyani, as well as a large proportion in Teshie. Thus, our three-treatment groups vary in terms of the amount of time they spend in the main catchment area of the SKY Girls program (which consists of selected neighborhoods of Accra). It is noted that girls in the last group (no treatment) may still have low exposure to some campaign components, for example, due to travel to the treatment area or through online access. This analysis is classified as an “intent-to-treat” analysis as we do not control for whether a particular girl actually received the treatment. The advantage of this method is that we avoid individual-level selection into treatment, as treatment status is based on geographical area rather than at the individual level. The resulting estimates can be viewed as the combined estimate of the reach of the program and the effectiveness of the program on those girls that it reached.

##### Dose-response analysis

The level of exposure to the SKY Girls program is also measured using a categorical variable representing awareness of the intervention (i.e., having ever heard of SKY Girls) and the number of SKY Girls components to which a girl reported exposure within the last 18 months. We hypothesize that girls who were exposed to a larger number of campaign components are more likely than other girls to experience changes in the key project outcomes measures. Our measure of program exposure includes four categories: (1) unaware of and unexposed to SKY Girls, (2) aware of SKY Girls but not exposed to any component, (3) exposed to 1–3 components, and (4) exposed to 4 or more components. These categories were defined based on the distribution of the sample and bivariate results that showed clear non-linearities in the association between the level of exposure and our key outcomes.

### Statistical analysis

For both approaches (i.e., intent-to-treat at the geographic level and dose-response), we use a difference-in-difference regression estimation method for panel data. We use a method that combines a fixed-effects model with inverse probability weighting by the Propensity Score (PS) [20, 26]. The resulting estimator is known as the doubly robust estimator [21] and can consistently estimate the treatment effect parameters as long as either the regression or the propensity score is correctly specified.

We calculate the propensity score with a probit regression where the dependent variable is a dummy indicating exposure to SKY Girls’ activities (e.g., living in Accra) and the independent variables are a set of *baseline* characteristics of the respondents. To ensure comparability between treated and untreated observations, participants outside the common support area (i.e., the area of overlap between the minimum and maximum values of the probability of exposure in both exposed and unexposed groups) were excluded from the regressions.

Based on the estimated propensity scores, weights were generated to obtain the two principal versions of aggregated treatment effects: the average treatment effect (ATE) and the average treatment effect on the treated (ATT). Since large weights can increase the variability of the estimated treatment effects, weights were truncated at a threshold determined by quantiles of the distribution (e.g., 1st and 99th percentile) [1].

To further ensure proper accounting for confounders, the regressions include controls for measurable time-variant characteristics, such as wealth, cellphone access, daily access to media channels, access to social media, participation in social activities, and exposure to other anti-smoking campaigns. As is standard in differences-in-differences models, our regressions also include dummy variables that indicate whether the observation is recorded during the post-intervention period, whether the observation is in the treated area, and whether the observation is in both the post-intervention period and the treated area.

All analyses were conducted using the *pscore*, *psmatch2* and *clogit* commands in Stata 15.0. Because coefficients from a logit model are not directly interpretable, we used the margins, dydx(*) command to obtain the marginal effect of exposure to SKY Girls on each outcome. The marginal effect represents the percentage point difference in outcomes for treated relative to comparison groups.

## Results

### Sample characteristics

There was no significant variation between the baseline and endline samples in terms of main socio-demographic variables measured, such as the girls’ age group, religion, household wealth quintile, and the average number of people living in their household (Table 3). There was variation in the geographic distribution of the sample across survey waves, with a slightly higher proportion of girls sampled in the endline survey living and studying in Accra (10% at baseline and 14% at endline). Access to cellphones, social media use and participation in social activities were similar in the baseline and endline samples. Approximately two thirds of girls sampled at both baseline and endline reported having access to a cellphone, and half reported having daily access to media channels. Social media use was low, with 22% of girls at baseline and 21% at endline using social media during the last week. Exposure to antismoking messages was similar between survey rounds, with an average of 60% of girls reporting having seen at least one message about the harms of smoking in the last month. Finally, the extent to which girls were in close contact with smokers was consistent across survey rounds; approximately 15% of girls declared that at least one close friend smoked, with slightly more girls (20%) having a close family member who smoked.

**Table 3:** Differences in baseline characteristics by sample composition in each survey round

	Baseline survey (*n* = 3775)	Endline survey (*n* = 2625)	*p*-value
School and household location			0.000
Living and studying in Accra	9.9	14.1	
Living or studying in Accra	15.9	9.7	
Living and studying in Teshie, Sunyani or Kumasi	74.2	76.2	
Age			0.248
13	31.6	33.2	
14	25.0	25.8	
15	20.6	19.9	
16	22.8	21.1	
Religion			0.251
Orthodox Christian (not catholic)	21.5	21.6	
Pentecostal/ Charismatic	50.4	49.1	
Muslim	18.5	20.3	
Other	9.6	8.9	
Household wealth quintile			0.824
Poorest	20.6	20.8	
Poor	21.1	21.9	
Middle	19.5	19.5	
Rich	19.4	19.5	
Richest	19.4	18.3	
Cellphone access			0.731
None	37.3	36.9	
Access to a cellphone	62.7	63.1	
Daily access to media channels			0.677
No daily access	49.8	49.2	
Access to at least one^a^	50.3	50.8	
Use of social media during the last week			0.388
No access	77.7	78.6	
Access to at least one^b^	22.3	21.4	
Number of monthly social activities^c^			0.853
0–1 activities	24.5	25.0	
2–3 activities	46.6	46.6	
4–5 activities	28.9	28.4	
Exposure to antismoking messages in different media^d^			0.732
None	40.2	39.8	
Yes, in 1–2 media	40.2	39.7	
Yes, in 3–5 media	19.7	20.5	
Close friends smoking			0.753
None	84.4	84.7	
At least one	15.6	15.3	
Close family members smoking			0.516
None	79.4	80.1	
At least one	20.6	19.9	

^a^Media channels include: television, radio. Newspaper, internet on a computer and internet on a mobile phone

^b^Social media include: Facebook, Twitter, Snapchat, Instagram, WhatsApp and YouTube

^c^Social activities such as going to bars/ drinking spots, restaurants, mall, funfairs and sporting events

^d^The media channels included are: television, radio, posters, magazine and social media

### Awareness and exposure to SKY Girls

As expected, awareness of SKY Girls (i.e., having ever heard of the program) was highest among girls in the full treatment group, followed by those in the partial treatment group (Table 4). Among girls living and studying in Accra, 90.5% were aware of SKY Girls, compared to 69.8% for those either living or studying in Accra. By contrast, the likelihood of having heard of the intervention was significantly lower among those in the comparison cities or untreated group (23%).

**Table 4:** Awareness of and exposure to specific SKY Girls components, by intention to treat status

Treatment status	Any awareness of SKY Girls	Exposure to specific components						Average number of components exposed to	n
		Magazine	Radio	School visits	Movie	Social media	Song		
Full treatment^a^	90.5	72.9	9.8	55.3	61.8	8.7	16.8	2.8	359
Partial treatment^b^	69.8	50.6	10.6	25.1	36.5	13.7	10.2	1.8	255
No treatment^c^	22.8	12.0	2.2	4.4	6.9	3.1	1.7	0.3	2001

^a^Girls living and studying in Accra

^b^Girls living or studying in Accra

^c^Girls living and studying in Kumasi, Sunyani or Teshie

Although girls may be aware of the SKY Girls program, they may not have been exposed to campaign messages or participated in SKY Girls activities. When analyzing the direct exposure to and/or participation in specific components of the intervention, we observe important differences across treatment groups. Among girls in the full treatment group, 72.9% read at least one SKY magazine, versus 50.6% for the partial treatment group and 12% for the untreated. Similarly, 61.8% of girls in the full treatment group watched at least one of the SKY movies (compared to 36.5 and 6.9%, respectively); 55.3% were present for a SKY Girls school visit (versus 25.1 and 4.4%), 9.8% listened to the SKY Live radio program (versus 10.6 and 2.2%); 8.7% follow SKY Girls on social media (versus13.7 and 3.1%), and 16.8% listened to the SKY Girls song (versus 10.2 and 1.7%).

Among girls in the full treatment group, the percentage who had heard of SKY also varied by demographic characteristics (Table 5). The highest rates of SKY Girls awareness were reported among 16 year-olds (69.6%), Muslims (88.8%), those in the richest wealth quintile (78.1%), those with access to a cell phone (70.8%), daily access to media (72.0%), recent use of social media (79.2%), and those who participated in several social activities (4–5 social events in the last month, 72.8%).

**Table 5:** Awareness of and exposure to SKY Girls by sociodemographic characteristics, among girls living and studying in Accra

	Any awareness of SKY Girls	Exposure by SKY component (a)						Average number of components exposed to	n
		Magazine	Radio	School visit	Movie	Social media	Song		
Age									
13	88.2	65.4	6.5	47.7	56.9	4.6	8.5	2.5	153
14	94.0	83.0	11.0	65.0	69.0	9.0	7.0	3.2	100
15	88.3	75.0	16.7	58.3	53.3	11.7	5.0	2.9	60
16	92.9	73.2	8.9	55.4	71.4	16.1	1.8	3.0	56
Religion									
Orthodox Christian (not catholic)	89.9	71.9	7.9	57.3	64.0	10.1	6.7	2.8	89
Pentecostal/ Charismatic	88.5	69.6	6.8	56.5	60.7	4.7	6.3	2.6	191
Muslim	94.5	82.2	16.4	45.2	63.0	13.7	5.5	3.3	73
Other	100.0	75.0	25.0	75.0	56.3	25.0	12.5	3.7	16
Household wealth quintile									
Poorest	92.7	74.4	7.3	52.4	62.2	1.2	7.3	2.8	82
Poor	96.7	75.4	3.3	67.2	68.9	6.6	8.2	2.9	61
Middle	84.7	67.1	15.3	45.9	56.5	11.8	4.7	2.6	85
Rich	87.3	73.2	5.6	56.3	60.6	9.9	8.5	2.9	71
Richest	92.9	75.7	15.7	58.6	62.9	14.3	4.3	3.1	70
Cellphone access									
None	88.2	69.6	5.2	52.6	57.8	3.0	6.7	2.5	135
Access to a cellphone	91.9	74.8	12.4	56.8	64.1	12.0	6.4	3.1	234
Daily access to media channels									
No daily access	89.5	69.6	10.5	51.5	62.6	7.6	4.7	2.7	171
Access to at least one	91.4	75.8	9.1	58.6	61.1	9.6	8.1	3.0	198
Use of social media during the last week									
No access	89.4	70.3	7.9	53.1	58.8	3.6	6.9	2.6	303
Use of at least one	95.5	84.9	18.2	65.2	75.8	31.8	4.6	3.8	66
Monthly participation in social activities									
0–1 activities	82.0	55.7	3.3	52.5	55.7	0.0	1.6	2.2	61
2–3 activities	91.2	74.9	8.2	53.2	57.9	8.8	8.2	2.7	171
4–5 activities	93.4	78.1	14.6	59.1	69.3	12.4	6.6	3.3	137

### Differences in outcome indicators by survey round and intention to treat

Table 6 shows trends in the outcome indicators for each of the three geographical treatment groups. The first panel shows indicators of the extent to which girls feel part of a supportive ‘sistahood.’ The findings indicate that being in the SKY Girls full treatment area is associated with increased perceptions of support. When compared to girls in the no treatment group, girls in the full treatment group (i.e., those living and studying in Accra) reported larger percentage point (pp) increases in perceived support from outside the family (5.1 pp. versus − 2.3 pp) and in perceived supported decisions by friends (10.0 pp. versus 4.1 pp).

**Table 6:** Differences in outcome indicators by survey round and intention to treat

Outcome indicator	Survey round	Treatment Status					
		Full treatment^a^		Partial treatment^b^		No treatment^c^	
		%	Diff.	%	Diff.	%	Diff.
1. Girls are part of supportive ‘sistahood’							
• Perception of sources of social support outside the family	Baseline	64.5	5.1	64.2	2.6	67.2	−2.3
	Endline	69.6		66.8		64.9	
• Perception that friends support their decisions	Baseline	29.8	10.0	34.6	8.1	33.9	4.1
	Endline	39.8		42.7		38.0	
• Perception of acceptance when saying no to friends	Baseline	59.9	15.7	62.2	8.9	57.1	15.5
	Endline	75.6		71.1		72.6	
• Perception of pressure to smoke shisha	Baseline	46.3	12.8	53.9	8.9	38.3	22.9
	Endline	59.1		62.8		61.2	
• Perception of pressure to smoke cigarettes	Baseline	69.6	−5.9	70.5	−0.5	64.6	2.7
	Endline	63.7		70.0		67.3	
2. Girls connect, share, explore issues							
• Held conversations with friends about smoking	Baseline	21.7	9.3	28.3	−8.9	21.2	2.1
	Endline	31.0		19.4		23.3	
• Held conversations with adults about smoking	Baseline	13.3	3.0	13.8	0.4	12.2	2.0
	Endline	16.3		14.2		14.2	
3. Girls express themselves through choices they make							
• Perception of being able to make choices for themselves	Baseline	87.5	5.2	90.6	0.7	87.5	1.9
	Endline	92.7		91.3		89.4	
4. Girls model refusal skills							
• Perception of confidence when saying no to friends	Baseline	90.8	3.2	94.9	1.1	89.7	6.2
	Endline	94.0		96.0		95.9	
5. Reduced aspiration to smoke							
• Agreement with beliefs that favor smoking shisha	Baseline	10.6	30.6	11.8	21.9	9.7	26.1
	Endline	41.2		33.7		35.8	
• Agreement with beliefs that favor smoking cigarettes	Baseline	44.7	3.3	48.2	−6.2	50.1	−7.0
	Endline	48.0		42.0		43.1	
• Agreement with the idea that peers can justify smoking shisha	Baseline	58.0	−17.1	57.6	−17.2	45.0	5.1
	Endline	40.9		40.4		50.1	
• Agreement with the idea that peers can justify smoking cigarettes	Baseline	47.2	−16.3	45.5	−9.4	48.5	−6.9
	Endline	30.9		36.1		41.6	
• Perception that most peers smoke tobacco	Baseline	48.8	7.6	57.5	12.9	54.8	12.1
	Endline	56.4		70.4		66.9	
• Perception that most peers smoke shisha	Baseline	30.4	26.0	42.9	22.7	28.8	30.0
	Endline	56.4		65.6		58.8	

^a^Girls living and studying in Accra

^b^Girls living or studying in Accra

^c^Girls living and studying in Kumasi, Sunyani or Teshie

Although perceived pressure to smoke shisha increased in all groups, this increase was weaker in the full treatment area than elsewhere. Specifically, girls in the no treatment group increased their perception of pressure to smoke by 22.9 pp., while girls in the full treatment group increased by only 12.8 pp. Moreover, while girls in the no treatment group increased their perceived pressure to smoke cigarettes by 2.7 pp., girls in the full treatment group decreased their perceived pressure to smoke by 5.9 pp. Being in the SKY treatment area does not appear to be associated with changes in the perception of acceptance when saying no to friends.

Results in the second panel of Table 6 suggest that the SKY program may have helped girls connect and explore issues. Girls in the full treatment group reported larger increases in conversations with friends about smoking than the control group (9.3 pp. versus 2.1 pp). Not surprisingly, the program appears to have little effect on conversations with parents about smoking (3.0% versus 2.0%).

As shown in the third and fourth panel, at baseline roughly nine out of ten girls in all three treatment groups already felt they were able to make choices for themselves and felt confident saying no to friends. Despite these high baseline levels, girls’ perceived ability to make choices for themselves increased slightly more rapidly in the full treatment group than in the control group (5.2 pp. versus 1.9 pp., see panel 3). While confidence to say no to friends increased somewhat in both the full treatment and partial treatment groups, it did so less rapidly than in the control group.

The last panel in Table 6 shows indicators of the extent to which the SKY Girls program may have helped strip the aspiration out of smoking. Unexpectedly, the results show more rapid increases in beliefs that favor smoking shisha and cigarettes in the treatment area than in the comparison areas. However, SKY Girls appears to have made strides in reducing the percentage of girls who agree that peers can justify smoking shisha (− 17.1 pp. for full treatment vs 5.1 pp. for control) or cigarettes (7.6 pp. versus 12.1 pp). Although smoking prevalence tend to increase with age [7], the perception that most peers smoke tobacco increased somewhat slower in the SKY full treatment area than in the no treatment area (7.6 pp. versus 12.1 pp). Similarly, the perception that most peers smoke shisha increased less in the treatment area (26.0 pp. versus 30.0 pp).

### Multivariate analysis

After the weights were generated based on the propensity scores, balance in the covariates between treated versus untreated girls was assessed using standardized differences. The standardized differences in the unweighted and weighted samples are presented in the Appendix, and for both analytical approaches (Tables 9 and 10). In the unweighted sample, the largest standardized difference was 0.59. In the weighted sample, all standardized differences were smaller than 0.10, confirming that the inverse probability weighting by the propensity score procedure resulted in samples in which the means of the covariates in the treated and comparison groups were very similar at baseline.

#### Intent-to-treat analysis at the geographic level

The first set of multivariate analyses used the fixed-effects regression models with inverse probability weighting by the propensity score and balanced samples to compare the full treatment and partial treatment groups with the no treatment group (Table 7). The average treatment effects on the treated (ATT) are presented as our main results. Results for the average treatment effect (ATE) are shown in Appendix Table 11. Girls living in the city of Accra, and those exposed to SKY Girls, are different from girls living in other more traditional or conservative cities in Ghana (e.g., Sunyani and Kumasi). When compared to the rest of Ghana, Accra is the largest city in the country, with high access to internet, high rates of immigration, less proportion of Muslim families, and with the infrastructure for conducting concerts/events in big malls and public venues. Therefore, our results aim to describe the effect that the program would have when implemented in similar contexts, making the ATT the most relevant result.

**Table 7:** Multivariate results (ATT) based on intent-to-treat analysis (base category = “no treatment”)

Outcome	Outcome indicator	Full treatment		Partial treatment	
		Marginal Effect	Std Err	Marginal Effect	Std Err
1. Girls are part of supportive ‘sistahood’	Perception of sources of social support outside the family	0.114*	−0.047	0.0598	−0.052
	Perception that friends support their decisions	0.0816	−0.044	0.0588	− 0.044
	Perception of acceptance when saying no to friends	0.0144	−0.04	− 0.0608	− 0.047
	Perception of pressure to smoke shisha (−)	− 0.0692	− 0.039	− 0.135**	− 0.049
	Perception of pressure to smoke cigarettes (−)	− 0.117*	− 0.047	−0.0652	− 0.055
2. Girls connect, share, explore issues	Held conversations with friends about smoking	0.123**	−0.043	−0.167**	− 0.062
	Held conversations with adults about smoking	0.0556	−0.061	0.0195	−0.071
3. Girls express themselves through choices they make	Perception of being able to make choices for themselves	0.117*	−0.056	− 0.0199	− 0.067
4. Girls model refusal skills	Perception of confidence when saying no to friends	−0.0844	− 0.079	− 0.152**	−0.059
5. Reduced aspiration to smoke	Agreement with beliefs that favor smoking shisha^a^	0.0423	−0.044	−0.0287	− 0.053
	Agreement with beliefs that favor smoking cigarettes^a^	0.129**	−0.04	0.0248	−0.049
	Agreement with the idea that peers can justify smoking shisha^a^	−0.203**	− 0.041	− 0.204**	−0.051
	Agreement with the idea that peers can justify smoking cigarettes^a^	−0.121**	− 0.044	− 0.0326	− 0.051
	Perception that most peers smoke tobacco (−)	− 0.0308	−0.041	0.0262	−0.046
	Perception that most peers smoke shisha (−)	−0.0309	−0.039	− 0.0382	−0.037

*Note*: 55 girls were deleted from the analysis due to uncommon support

^a^Effects on each of the independent components of the index are presented in the Appendix

** *p* < 0.01, * *p* < 0.05

Multivariate results are largely consistent with the observed trends in the outcome indicators for treated and untreated girls. After controlling for other factors, we find that the SKY Girls program had some success in creating a supportive ‘sistahood’ for young girls. Specifically, among girls in the full treatment group, we found: (i) an 11.4 pp. increase (95% CI [2.1, 20.7]) in perceptions of having social support outside the family; (ii) an 11.7 pp. decrease (95% CI [− 20.8, − 2.6]) in feeling pressure to smoke cigarettes. The program does not appear to have affected the other indicators of having a supportive ‘sistahood’.

There is also evidence that the program helped connect girls and increased conversations about smoking among them. The results show that there was a 12.3 pp. increase (95% CI [3.8, 20.8]) in having conversations with friends about smoking. The finding that the partial treatment group experienced a decline in conversations with friends about smoking, relative to the control group, suggests that repeated program exposure may be needed to succeed in empowering girls to have such discussions among themselves. As expected from the bivariate trends, SKY Girls did not have an effect on the likelihood that girls spoke with their parents about smoking. The latter is consistent with the program’s focus on girls’ empowerment and peer support.

SKY girls had a notable effect on girl’s ability to express themselves through the choices they make. The findings show an 11.7 pp. increase (95% CI [0.6, 22.8]) in the perceived self-efficacy to make choices for one’s self among girls in the full treatment group relative to the control group. However, there is no evidence that the program was able to strengthen girl’s confidence that they can say no to friends. This is an area that may merit more attention during subsequent phases of the program.

Finally, there is mixed evidence about SKY Girls’ ability to reduce young girls’ aspiration to smoke. As shown in the bottom panel of Table 6, the program has been quite successful in reducing girl’s perception that peers can justify smoking. Specifically, we found a 12.1 pp. decrease (95% CI [− 20.7, − 3.5]) in agreement with the idea that peers can justify smoking cigarettes. In addition, there was a 20.3 pp. decrease (95% CI [− 28.4, − 12.2]) in agreement with the idea that peers can justify smoking shisha in the treatment group, and a 20.4 pp. decline (95% CI [− 30.3, − 10.5]) for the partial treatment group.

However, the program did not succeed in reducing beliefs that favor smoking. Contrary to expectations, girls in the full treatment group exhibited a 12.9 pp. increase (95% CI [5.0, 20.8]) in the agreement with beliefs that favor smoking cigarettes, and there was no effect on beliefs that favor smoking shisha. Likewise, the program was unable to affect girls’ perceptions that most peers smoke tobacco or shisha.

#### Dose-response analysis

The second set of estimations examined the dose-response effects of the level of exposure to SKY Girls’ components (Table 8). Again we present results for the average treatment effect on the treated (ATT); results for the average treatment effect are shown in Appendix Table 12. Intuitively, we expected that for most of the indicators affected by the SKY Girls program, the effect would increase with the level of exposure. Our results show that this is not always the case.

**Table 8:** Multivariate results (ATT) based on the dose-response analysis (base category = unaware and unexposed to SKY)

Outcome	Outcome indicator	Exposed to 4+		Exposed to 1–3		Unexposed but aware	
		Marginal Effect	Std Err	Marginal Effect	Std Err	Marginal Effect	Std Err
1. Girls are part of supportive ‘sistahood’	Perception of sources of social support outside the family	0.016	−0.801	0.016	− 0.039	− 0.019	− 0.04
	Perception that friends support their decisions	0.094	−0.083	0.009	−0.041	0.047	−0.051
	Perception of acceptance when saying no to friends	0.012	−0.826	−0.017	− 0.034	− 0.066	− 0.064
	Perception of pressure to smoke shisha (−)	− 0.071	− 0.213	− 0.017	− 0.034	0.054	− 0.048
	Perception of pressure to smoke cigarettes (−)	− 0.096	− 0.106	− 0.028	− 0.04	0.038	− 0.059
2. Girls connect, share, explore issues	Held conversations with friends about smoking	0.087	− 0.128	0.041	− 0.039	0.002	− 0.061
	Held conversations with adults about smoking	0.124*	−0.048	− 0.053	− 0.055	− 0.021	− 0.078
3. Girls express themselves through choices they make	Perception of being able to make choices for themselves	0.293*	−0.014	0.130*	−0.051	0.095	−0.088
4. Girls model refusal skills	Perception of confidence when saying no to friends	0.234**	− 0.006	−0.047	− 0.047	− 0.181	− 0.128
5. Reduced aspiration to smoke	Agreement with beliefs that favor smoking shisha^a^	−0.014	− 0.811	0	− 0.037	− 0.069	−0.074
	Agreement with beliefs that favor smoking cigarettes^a^	0.07	−0.171	0.034	−0.039	0.008	−0.057
	Agreement with the idea that peers can justify smoking shisha^a^	−0.128*	− 0.016	− 0.0950**	− 0.036	0.033	−0.055
	Agreement with the idea that peers can justify smoking cigarettes^a^	−0.045	− 0.413	− 0.025	−0.038	0.063	−0.056
	Perception that most peers smoke tobacco (−)	0.033	−0.58	0.019	−0.032	0.056	−0.064
	Perception that most peers smoke shisha (−)	0.038	−0.378	0.058	−0.037	0.019	−0.056

*Note*: 18 girls were deleted from the analysis due to uncommon support

^a^Effects on each of the independent components of the index are presented in the Appendix

** *p* < 0.01, * *p* < 0.05

Although the previous multivariate results showed evidence that the program improved perceived social support outside the family and reduced perceived pressure to smoke cigarettes, there are no significant dose-response effects for either of these variables (Table 8, first panel). This suggests that these outcomes can be improved by means of just one of two program components that have wide reach among the target group.

The results for the fixed-effects logit models with balanced samples further show that although SKY Girls improved the extent to which young girls talked with friends about smoking, there is no dose-response effect. More importantly, even though overall the program had no effect on the extent to which girls spoke with adults about smoking, the dose-response analysis show that girls exposed to four or more SKY Girls program components had a 12.4 pp. increase (95% CI [3.0, 21.8]) in conversations with adults about smoking relative to girls unexposed to SKY. The finding that there was no such effect for girls exposed to 1–3 SKY Girls program components implies that improving conversations with parents and other adults requires repeated program exposure. Hence improving conversations with parents and other adults is likely to require more programmatic effort, and most likely also a longer intervention period.

Table 8 also shows that there are significant dose-response effects for both girls’ ability to express themselves through the choices they make, and their ability to model refusal skills. Specifically, we found a 29.3 pp. increase (95% CI [26.6, 32.0]) in autonomy in making choices for themselves for girls exposed to at least four program components. The finding there is also a 13.0 pp. increase (95% CI [3.0, 23.0]) for girls exposed to 1–3 program components indicates that even modest programmatic effort can help improve girls’ ability to make choices.

Although there is no evidence that overall SKY Girls was able to affect girls’ self-efficacy to oppose or refuse friends, there are significant dose effects. We found a 23.4 pp. increase (95% CI [22.2, 24.6]) in the perceived confidence of saying no to friends for girls exposed to four or more program components, but no effect for girls with lower levels of exposure. Once again, this suggests that self-efficacy to oppose friends is more difficult to change, and therefore likely to require a longer intervention period.

As shown in the last panel of Table 8, there are clear dose effects only for one of the five measures of the aspiration to smoke. The dose effects for agreement with the idea that peers can justify smoking shisha show stronger effects (12.8 pp. reduction (95% CI [− 15.9, − 9.7]) for girls exposed to four or more SKY components relative to a 9.5 pp. decrease (95% CI [− 16.6, − 2.4]) for those exposed to 1–3 components). There are no dose effects for the other four indicators of the aspiration to smoke.

## Discussion

The present study is the first impact evaluation of SKY Girls, a youth-focused smoking-prevention and empowerment campaign targeting girls in Ghana. Consistent with its multiple-channel exposure strategy, 90.5% of the girls in the full treatment group (i.e., those living and studying in the intervention city, Accra) were aware of the intervention. Multivariate results show that the program was able to achieve improvements in many key outcome indicators. Some of these improvements required more intensive exposure than others. Perceived social support outside the family, perceived pressure to smoke cigarettes and talking with friends about smoking appear to be affected by any level of exposure to the SKY Girls program. On the other hand, indicators measuring autonomy and adult interactions required repeated exposures or exposures to multiple SKY Girls components. These indicators included talking with adults about smoking, having the ability to make choices for one’s self, and modeling refusal skills.

These encouraging results are consistent with evaluations of similar campaigns to reduce smoking behaviors. For example, Brendryen et al. [3] documented that the use of multi-channel, automated and interactive digital media had positive and long-term effects on smoking abstinence rates and on the level of post-cessation self-efficacy. Similarly, Baskerville et al. [2] showed that a multicomponent web-based and social media approach increased higher 7-day and 30-day smoking quit rates among youth.

Although most results were in the expected direction, we observed some counter-intuitive or unexpected results. When analyzing beliefs that favor smoking cigarettes, girls in comparison cities (i.e., those with no treatment) had a greater reduction in their agreement with beliefs that favor smoking cigarettes than girls in the group with full treatment. Further studies, ideally qualitative, are needed to better understand this phenomenon.

### Strengths and limitations

A key strength of this evaluation is the use of panel data to eliminate the risk of individual confounding factors and temporality of causality. However, this evaluation was not a randomized control trial; therefore, the possibility that time variant confounding factors could have affected these results still exists. Another limitation is the use of self-reported exposure outcomes susceptible to social desirability and interviewer biases. It is possible that exposure to the SKY Girls program prompts respondents to report decreased desirability of smoking to the interviewer, rather than actually feeling it as less desirable. Because smoking rates among girls in the study population are still very low, we are unable to measure if the effect of the observed improvements in key program outcome measures has resulted in changes in smoking rates (i.e., the variance in the smoking rates is too low to test this). Future longitudinal studies should consider following the girls in the study population at least until the average age at smoking uptake.

## Conclusion

SKY Girls Ghana aimed to reduce attitudes favoring tobacco use and to reduce intentions to use tobacco by increasing broader resilience and empowering young girls. Based on the results of this impact evaluation, we conclude that the intervention was effective in increasing perceived support outside the family, decreasing the self-reported pressure to smoke cigarettes, increasing conversations with friends about smoking, improving the ability of girls to make decisions for themselves, and decreasing agreement with the idea that peers can justify smoking.

## Recommendations

The success of SKY Girls in empowering, providing perceived support and helping young girls make choices can have important implications for other adolescent health issues. Girls’ empowerment is known to affect a wide range of health issues, such as early sex, alcohol use, and dating violence. Hence, these results suggest that the SKY Girls approach for empowering girls – through a combination of in-school, community-based, mass media, and social media activities – can potentially serve as a roadmap to be adapted for use in other settings.

### Supplementary Information

**Additional file 1.** Endline Questionnaire SKY Girls.

## Data Availability

The datasets analyzed during the current study are available from the corresponding author on reasonable request.

## Appendix

**Table 9:** Reporting of balance assessment, intention-to-treat analysis

Specification	Variable	Sample	ATT			ATE		
			Mean treatment group	Mean control group	Std-difference	Mean treatment group	Mean control group	Std-difference
Full intention to treat vs. no intention to treat	Cellphone access	Unweighted	0.64	0.62	0.03	0.64	0.62	0.03
		Weighted	0.64	0.64	0.00	0.63	0.62	0.01
	Daily access to media channels	Unweighted	0.68	0.64	0.06	0.68	0.64	0.06
		Weighted	0.68	0.68	0.00	0.66	0.65	0.02
	Monthly participation in social activities	Unweighted	2.94	2.40	0.40	2.94	2.40	0.40
		Weighted	2.94	2.95	0.00	2.65	2.48	0.12
	Use of social media during the last week	Unweighted	0.27	0.36	−0.11	0.27	0.36	−0.11
		Weighted	0.27	0.26	0.02	0.31	0.32	−0.02
	Low wealth quintile	Unweighted	0.17	0.23	−0.15	0.17	0.23	−0.15
		Weighted	0.17	0.17	0.00	0.20	0.22	−0.04
	Middle wealth quintile	Unweighted	0.23	0.19	0.08	0.23	0.19	0.08
		Weighted	0.23	0.23	0.00	0.21	0.20	0.02
	High wealth quintile	Unweighted	0.19	0.19	0.00	0.19	0.19	0.00
		Weighted	0.19	0.19	0.01	0.19	0.19	0.00
	Highest wealth quintile	Unweighted	0.19	0.18	0.02	0.19	0.18	0.02
		Weighted	0.19	0.19	0.00	0.19	0.18	0.01
	Number of antismoking messages exposed to	Unweighted	1.39	1.21	0.13	1.39	1.21	0.13
		Weighted	1.39	1.38	0.01	1.28	1.23	0.04
Partial intention to treat vs. no intention to treat	Cellphone access	Unweighted	0.64	0.62	0.04	0.64	0.62	0.04
		Weighted	0.64	0.64	0.00	0.63	0.62	0.02
	Daily access to media channels	Unweighted	0.71	0.64	0.09	0.71	0.64	0.09
		Weighted	0.71	0.70	0.01	0.66	0.65	0.00
	Monthly participation in social activities	Unweighted	3.22	2.40	0.59	3.22	2.40	0.59
		Weighted	3.21	3.18	0.02	2.63	2.56	0.05
	Use of social media during the last week	Unweighted	0.59	0.36	0.23	0.59	0.36	0.23
		Weighted	0.59	0.58	0.01	0.42	0.40	0.02
	Low wealth quintile	Unweighted	0.15	0.23	−0.19	0.15	0.23	−0.19
		Weighted	0.15	0.15	0.01	0.20	0.21	−0.03
	Middle wealth quintile	Unweighted	0.18	0.19	− 0.03	0.18	0.19	− 0.03
		Weighted	0.18	0.19	−0.03	0.21	0.19	0.04
	High wealth quintile	Unweighted	0.20	0.19	0.02	0.20	0.19	0.02
		Weighted	0.20	0.20	0.01	0.19	0.20	−0.01
	Highest wealth quintile	Unweighted	0.26	0.18	0.19	0.26	0.18	0.19
		Weighted	0.26	0.26	0.01	0.20	0.20	0.01
	Number of antismoking messages exposed to	Unweighted	1.35	1.21	0.10	1.35	1.21	0.10
		Weighted	1.35	1.32	0.02	1.21	1.23	−0.02

**Table 10:** Reporting of balance assessment, dose-response analysis

Specification	Variable	Sample	ATT			ATE		
			Mean treatment group	Mean control group	Std-difference	Mean treatment group	Mean control group	Std-difference
4 + components vs. unexposed	Cellphone access	Unweighted	0.68	0.62	0.12	0.68	0.62	0.12
		Weighted	0.68	0.68	0.01	0.68	0.63	0.12
	Daily access to media channels	Unweighted	0.69	0.65	0.06	0.69	0.65	0.06
		Weighted	0.69	0.69	0.00	0.69	0.65	0.05
	Monthly participation in social activities	Unweighted	2.66	2.58	0.06	2.66	2.58	0.06
		Weighted	2.66	2.65	0.01	2.66	2.58	0.06
	Use of social media during the last week	Unweighted	0.54	0.38	0.17	0.54	0.38	0.17
		Weighted	0.54	0.51	0.03	0.54	0.38	0.18
	Low wealth quintile	Unweighted	0.23	0.21	0.04	0.23	0.21	0.04
		Weighted	0.23	0.23	0.00	0.23	0.21	0.04
	Middle wealth quintile	Unweighted	0.19	0.19	−0.01	0.19	0.19	− 0.01
		Weighted	0.19	0.19	0.00	0.19	0.19	−0.01
	High wealth quintile	Unweighted	0.18	0.19	−0.05	0.18	0.19	−0.05
		Weighted	0.18	0.17	0.00	0.17	0.19	−0.05
	Highest wealth quintile	Unweighted	0.20	0.19	0.02	0.20	0.19	0.02
		Weighted	0.20	0.20	0.00	0.20	0.19	0.03
	Number of antismoking messages exposed to	Unweighted	1.46	1.24	0.16	1.46	1.24	0.16
		Weighted	1.46	1.43	0.02	1.46	1.24	0.16
1–3 components vs. unexposed	Cellphone access	Unweighted	0.63	0.63	0.00	0.63	0.63	0.00
		Weighted	0.62	0.63	0.00	0.63	0.63	0.01
	Daily access to media channels	Unweighted	0.67	0.65	0.03	0.67	0.65	0.03
		Weighted	0.68	0.67	0.00	0.65	0.65	0.00
	Monthly participation in social activities	Unweighted	2.97	2.52	0.33	2.97	2.52	0.33
		Weighted	2.97	2.99	−0.01	2.67	2.59	0.06
	Use of social media during the last week	Unweighted	0.49	0.37	0.13	0.49	0.37	0.13
		Weighted	0.49	0.50	0.00	0.41	0.39	0.02
	Low wealth quintile	Unweighted	0.19	0.21	−0.06	0.19	0.21	− 0.06
		Weighted	0.19	0.19	0.00	0.21	0.21	− 0.01
	Middle wealth quintile	Unweighted	0.20	0.19	0.01	0.20	0.19	0.01
		Weighted	0.20	0.20	0.00	0.19	0.19	0.00
	High wealth quintile	Unweighted	0.17	0.20	−0.07	0.17	0.20	−0.07
		Weighted	0.17	0.17	0.00	0.19	0.19	0.00
	Highest wealth quintile	Unweighted	0.22	0.19	0.08	0.22	0.19	0.08
		Weighted	0.22	0.22	0.00	0.20	0.19	0.01
	Number of antismoking messages exposed to	Unweighted	1.37	1.23	0.10	1.37	1.23	0.10
		Weighted	1.37	1.37	0.00	1.27	1.25	0.01
Unexposed but aware vs. unexposed	Cellphone access	Unweighted	0.70	0.62	0.17	0.70	0.62	0.17
		Weighted	0.70	0.70	0.00	0.69	0.63	0.13
	Daily access to media channels	Unweighted	0.80	0.64	0.21	0.80	0.64	0.21
		Weighted	0.80	0.78	0.03	0.75	0.65	0.13
	Monthly participation in social activities	Unweighted	3.40	2.52	0.65	3.40	2.52	0.65
		Weighted	3.40	3.38	0.02	3.26	2.60	0.49
	Use of social media during the last week	Unweighted	0.60	0.38	0.24	0.60	0.38	0.24
		Weighted	0.60	0.58	0.02	0.55	0.38	0.18
	Low wealth quintile	Unweighted	0.16	0.22	−0.16	0.16	0.22	−0.16
		Weighted	0.16	0.16	0.00	0.16	0.20	−0.10
	Middle wealth quintile	Unweighted	0.18	0.20	−0.04	0.18	0.20	−0.04
		Weighted	0.18	0.18	0.00	0.19	0.20	−0.02
	High wealth quintile	Unweighted	0.24	0.19	0.11	0.24	0.19	0.11
		Weighted	0.24	0.23	0.01	0.23	0.19	0.08
	Highest wealth quintile	Unweighted	0.25	0.19	0.15	0.25	0.19	0.15
		Weighted	0.25	0.25	0.01	0.24	0.19	0.11
	Number of antismoking messages exposed to	Unweighted	1.64	1.22	0.29	1.64	1.22	0.29
		Weighted	1.64	1.60	0.03	1.50	1.25	0.18

**Table 11:** Multivariate results (ATE) based on the intention-to-treat analysis (base category = no treatment)

Outcome	Outcome indicator	Full treatment		Partial treatment	
		Marginal Effect	Std Err	Marginal Effect	Std Err
1. Girls are part of supportive ‘sistahood’	Perception of sources of social support outside the family	0.0926	(0.048)	0.0419	(0.054)
	Perception that friends support their decisions	0.0563	(0.044)	0.0749	(0.043)
	Perception of acceptance when saying no to friends	0.0037	(0.040)	− 0.0983*	(0.050)
	Perception of pressure to smoke shisha (−)	− 0.0706	(0.043)	− 0.134**	(0.051)
	Perception of pressure to smoke cigarettes (−)	− 0.0974*	(0.048)	− 0.0481	(0.057)
2. Girls connect, share, explore issues	Held conversations with friends about smoking	0.127**	(0.043)	−0.157*	(0.063)
	Held conversations with adults about smoking	0.0845	(0.060)	0.00484	(0.072)
3. Girls express themselves through choices they make	Perception of being able to make choices for themselves	0.115*	(0.057)	−0.0011	(0.068)
4. Girls model refusal skills	Perception of confidence when saying no to friends	−0.0722	(0.079)	−0.195**	(0.074)
5. Reduced aspiration to smoke	Agreement with beliefs that favor smoking shisha (a)	0.0513	(0.047)	−0.0445	(0.060)
	Agreement with beliefs that favor smoking cigarettes (a)	0.117**	(0.040)	0.0255	(0.055)
	Agreement with the idea that peers can justify smoking shisha (a)	−0.216**	(0.042)	−0.205**	(0.054)
	Agreement with the idea that peers can justify smoking cigarettes (a)	− 0.118**	(0.044)	−0.00162	(0.052)
	Perception that most peers smoke tobacco (−)	−0.0285	(0.044)	0.0286	(0.054)
	Perception that most peers smoke shisha (−)	−0.0239	(0.043)	−0.0527	(0.046)

** *p* <.01, * *p* <.05

**Table 12:** Multivariate results (ATE) based on the dose-response analysis (base category = unaware and unexposed to SKY)

Outcome	Outcome indicator	Exposed to 4+		Exposed to 1–3		Unexposed but aware	
		Marginal Effect	Std Err	Marginal Effect	Std Err	Marginal Effect	Std Err
1. Girls are part of supportive ‘sistahood’	Perception of sources of social support outside the family	0.028	(0.645)	0.044	(0.271)	−0.014	(0.767)
	Perception that friends support their decisions	0.069	(0.177)	0.008	(0.844)	0.046	(0.377)
	Perception of acceptance when saying no to friends	−0.021	(0.708)	− 0.021	(0.527)	−0.062	(0.315)
	Perception of pressure to smoke shisha (−)	−0.067	(0.237)	−0.026	(0.466)	0.076	(0.175)
	Perception of pressure to smoke cigarettes (−)	− 0.081	(0.163)	− 0.040	(0.325)	0.049	(0.412)
2. Girls connect, share, explore issues	Held conversations with friends about smoking	0.096	(0.104)	0.042	(0.296)	−0.004	(0.953)
	Held conversations with adults about smoking	0.144*	(0.021)	−0.065	(0.243)	−0.013	(0.868)
3. Girls express themselves through choices they make	Perception of being able to make choices for themselves	0.238**	(0.002)	0.120*	(0.019)	0.074	(0.366)
4. Girls model refusal skills	Perception of confidence when saying no to friends	0.165	(0.057)	−0.047	(0.334)	− 0.155	(0.195)
5. Reduced aspiration to smoke	Agreement with beliefs that favor smoking shisha (a)	−0.022	(0.713)	0.001	(0.982)	−0.072	(0.299)
	Agreement with beliefs that favor smoking cigarettes (a)	0.046	(0.354)	0.036	(0.356)	0.002	(0.975)
	Agreement with the idea that peers can justify smoking shisha (a)	−0.160**	(0.002)	−0.101**	(0.006)	0.031	(0.579)
	Agreement with the idea that peers can justify smoking cigarettes (a)	−0.076	(0.173)	−0.031	(0.424)	0.057	(0.303)
	Perception that most peers smoke tobacco (−)	0.029	(0.614)	0.023	(0.495)	0.050	(0.425)
	Perception that most peers smoke shisha (−)	0.052	(0.282)	0.068	(0.073)	0.017	(0.758)

** *p* <.01, * *p* <.05

## Abbreviations

ATE: Average Treatment Effect
ATT: Average Treatment Effect on the Treated
CI: Confidence Interval
Diff: Difference
EA: Enumeration Area
GB: Good Business
NAA: Now Available Africa
ORID: Office of Research, Innovation and Development
pp.: Percentage point
PPS: Probability proportional to size
PS: Propensity score
TOC: Theory of Change
U.S.: United States
