# Supplementary material for: Evaluation of a multimedia youth anti-smoking and girls’ empowerment campaign: SKY Girls Ghana
Source: BMC Public Health. 2020 Nov 17;20:1734. doi: 10.1186/s12889-020-09837-5 (PMC7670706; doi:10.1186/s12889-020-09837-5)
Supplement: Supplementary file 1 — Additional file 1. Endline Questionnaire SKY Girls. [file 12889_2020_9837_MOESM1_ESM.pdf]

Rapid assessment of knowledge, attitudes and perception of students aged 13 to 16 towards Smoking in the cities of Accra, Kumasi, Sunyani, Teshie

ENDLINE SURVEY

**City**	Accra ………………1<br>Kumasi ……………2<br>Sunyani …………..3<br>Teshie……………..4
**EA number**	
**Structure Number**	
**Household number**	
**Respondent's line no.**	
**Supervisor Code**	
**Field Assistant Code**	
**Date**	Day/Month/Year<br>_____ /_____ /_____
**HH GPS coordinates**	Latitude:<br>__ __. __ __ __ __ __ __
	Longitude:<br>__ __. __ __ __ __ __ __

**Please select the most appropriate:**	
The adolescent and his/her family were found in the same household	……1
The family and the adolescent have moved somewhere else	……2
The family is still in the household, but the adolescent has permanently moved	……3
The family is still in the household, but the adolescent is temporarily somewhere else	……4

Introduction

This survey asks you about how you feel about smoking. All your answers will be kept secret and won't be shown or shared to anyone outside the research team. It is very important to be as truthful and accurate as possible. All the information you provide will be kept confidential. Apart from the research team no one else can link you to your information and you will not be named in any reports.

HQ8.	Does your household have:		YES	NO
	b. A wall clock?	Wall Clock	1	0
	c. A radio?	Radio	1	0
	e. A color television?	Color Television	1	0
	f. A mobile telephone?	Mobile Telephone	1	0
	h. A refrigerator?	Refrigerator	1	0
	l. Computer/Tablet computer?	Computer/Tablet	1	0
	n. Video deck/DVD/VCD?	Video Deck/DVD/VCD	1	0
	o. Sewing machine?	Sewing Machine	1	0
	r. Cabinet/cupboard?	Cabinet/Cupboard	1	0
	s. Access to the Internet in any device?	Internet access	1	0
HQ10.	Does your household have?<br>e. A car or truck	Car/Truck	1	0

HQ11.	Does any member of this household have a Bank account?	Yes . . . . 1<br>No 0

BACKGROUND

Line number of the respondent in the household rooster ……… ………

No.	Question	Coding	Categories
Q1	Circle Sex of the respondent	Male<br>Female	…………………1<br>…………………2
Q2	How old are you in complete years? *(confirm there is not an inconsistency with the baseline age)*	13 years old<br>14 years old<br>15 years old<br>16 years old<br>17 years old<br>18 years old<br>19 years old	…………………1<br>…………………2<br>…………………3<br>…………………4<br>…………………5<br>…………………6<br>…………………7
N1	Do you live in Greater Accra?	Yes<br>No *(skip to N3)*	…………………1<br>…………………0
N2	If Yes to **N1**, In which of these neighbourhoods do you live?	Madina<br>Adenta<br>St Johns<br>Achimota<br>Spintex<br>Haatso<br>Legon<br>Dome<br>Other *(please state)*	…………………1<br>…………………2<br>…………………3<br>…………………4<br>…………………5<br>…………………6<br>…………………7<br>…………………8<br>…………………9

N3	Do you currently attend school, or have you attended school during the last year?	Yes<br>No *(skip to **Q8**)*	…………………1<br>…………………0
N4	If Yes to **N3**, what is the name of your school?	List attached below	
Q7	If Yes to **N3**, what is the highest grade that you have completed? which class are you in?		
N6	If Yes to **N3**, Is your school located in Greater Accra?		
N7	If Yes to **N6**, in which of these neighbourhoods is your school located?	Madina<br>Adenta<br>St Johns<br>Achimota<br>Spintex<br>Haatso<br>Legon<br>Dome<br>Other (please state)	…………………1<br>…………………2<br>…………………3<br>…………………4<br>…………………5<br>…………………6<br>…………………7<br>…………………8<br>…………………9
Q8	Do you own/have access to a mobile phone?	Yes, I own a mobile phone with internet<br>Yes, I own a mobile phone with no internet<br>I have access to a mobile phone which is not mine with internet<br>I have access to a mobile phone which is not mine without internet<br>No, I do not own or have access to a phone *(go to Q14)*	…………………1<br>…………………2<br>…………………3<br><br>…………………4<br><br>…………………0
Q9	If Yes to **Q8**, can you please provide the complete number (s)?	| | | | | | | | | | | <br>| | | | | | | | | | |	

			Yes, daily	Yes, at least once a week	No
Q14	Do you have access to the following media?	Television	……1	……2	……0
		Radio	……1	……2	……0
		Newspaper	……1	……2	……0
		Internet on a computer	……1	……2	……0

		Internet on a mobile phone	……1	……2	……0

Q16	In the past week, how often did you use/visit the following websites/apps?	Never (0)	1-2 times (1)	3-5 times (2)	Every day (3)
	a) Facebook	0	1	2	3
	b) Twitter	0	1	2	3
	c) Snapchat	0	1	2	3
	d) Instagram	0	1	2	3
	e) WhatsApp	0	1	2	3
	f) YouTube	0	1	2	3

TOBACCO KNOWLEDGE

No.	Question	Coding	Categories		
Q17	Which of the following do you think are tobacco products? *(Tick all that apply)* *Read out*		Yes	No	I don't know
		a) Cigarettes	……1	……0	……9
		b) Marijuana	……1	……0	……9
		c) Snuff	……1	……0	……9
		d) Betel leaves	……1	……0	……9
		e) Hash	……1	……0	……9
		f) Weed	……1	……0	……9
		g) Shisha	……1	……0	……9
		h) Chewing Tobacco	……1	……0	……9
		i) E-Cigarettes	……1	……0	……9
		j) Cigars	……1	……0	……9
		k) Khat	……1	……0	……9
Q18	Are there any health consequences from smoking cigarettes?<br>**If No or DK, skip to Q20		……1	……0	……9
Q19	What are these health consequences from smoking cigarettes? *(Tick all that apply)*	a) Coronary heart disease	……1	……0	……9
		b) Stroke	……1	……0	……9
		c) Lung cancer	……1	……0	……9
		d) Can affect the baby's health if a pregnant woman smoke	……1	……0	……9
		e) Other (specify) ……………………			

Q20	Are there any health consequences from smoking shisha?<br>**If No or DK, skip to Q22				
Q21	What are these health consequences from smoking shisha? *(Tick all that apply)*	a) Coronary heart disease<br>b) Stroke<br>c) Lung cancer<br>d) Can affect the baby's health if a pregnant woman smoke<br>e) Other (specify) …………………….	……1<br>……1<br>……1<br>……1	……0<br>……0<br>……0<br>……0	……9<br>……9<br>……9<br>……9

Q22	How much damage can smoking ________ cause to your health?<br><br>*Check **Q18** and **Q20**. If yes to either one, ask the appropriate corresponding question	It doesn't cause any damage (0)	Some damage but nothing irreversible (1)	Irreversible damage (2)	I don't know (9)
	a) Shisha				
	b) Cigarettes				

EXPOSURE TO MESSAGES ABOUT TOBACCO

			TV	Radio	Posters	Magazines	Social media
Q23	In the past month, have you seen or heard any messages about the **harms of smoking** on each of the following media? *(Tick all that apply)*	Yes, lots<br>Yes, some<br>No	…….0<br>…….1<br>…….2	…….0<br>…….1<br>…….2	…….0<br>…….1<br>…….2	…….0<br>…….1<br>…….2	…….0<br>…….1<br>…….2
T1	If Yes to **Q23**, was it a message from SKY Girls?	Yes<br>No<br>DK	…….1<br>…….0<br>…….9	…….1<br>…….0<br>……9	…….1<br>…….0<br>……9	…….1<br>…….0<br>……9	…….1<br>…….0<br>……9

Q24	In the past month, have you seen any **advertisements** for cigarettes or shisha?	No, none<br>Yes, some<br>Yes, lots			…………..0<br>…………..1<br>…………..2	
Q27	If Yes to **Q24**: how did the person (s) in the advertisement look like?	Strongly agree (1)	Agree (2)	Neutral (3)	Disagree (4)	Strongly disagree (5)
	a) Cool					
	b) Healthy					
	c) Happy					

	d) Young					
	e) Wealthy					

Q28	In the past month, have you seen anyone smoking in films, videos, TV or magazine pictures?	No, none<br>Yes, some<br>Yes, lots	............0<br>............1<br>............2
Q29	In the past month, have you seen pictures/videos of any of your friends smoking in social media?	No, none<br>Yes, some<br>Yes, lots	............0<br>............1<br>............2
Q30	If you attended school, were you taught in any of your classes about the consequences of smoking tobacco?	Yes<br>No<br>I don't know	............1<br>............0<br>............9
T2	If Yes to **Q30**, who provided the information? (read out all options)	A teacher<br>A doctor or nurse<br>A government official<br>SKY girls<br>A church<br>Others	............1<br>............2<br>..........3<br>............4<br>............5<br>..........6

BELIEFS AND ATTITUDES

No.	Questions	Categories and coding				
Q33	Please say whether you agree or disagree with each of the statements below. Please don't leave any out. *(Tick all that apply)*	Strongly agree (1)	Agree (2)	Neutr. (3)	Disagree (4)	Strongly disagree (5)
Positive beliefs towards smoking	a) Smoking cigarettes helps with stress	1	2	3	4	5
	b) Smoking cigarettes makes you confident	1	2	3	4	5
	c) Smoking cigarettes is enjoyable	1	2	3	4	5
	d) Smoking cigarettes makes you more intelligent	1	2	3	4	5
	e) Smoking cigarettes is cool	1	2	3	4	5
	f) People who smoke cigarettes are more popular	1	2	3	4	5
	g) Girls who smoke cigarettes are more attractive	1	2	3	4	5
	h) Boys who smoke cigarettes are more attractive	1	2	3	4	5
	i) Trying cigarettes is part of growing up	1	2	3	4	5
	j) Smoking shisha helps with stress	1	2	3	4	5
	k) Smoking shisha makes you confident	1	2	3	4	5
	l) Smoking shisha is enjoyable	1	2	3	4	5
	m) Smoking shisha makes you more intelligent	1	2	3	4	5
	n) Smoking shisha is cool	1	2	3	4	5
	o) People who smoke shisha are more popular	1	2	3	4	5

	p) Girls who smoke shisha are more attractive	1	2	3	4	5
	q) Boys who smoke shisha are more attractive	1	2	3	4	5
	r) Trying shisha is part of growing up	1	2	3	4	5
Negative/neutral beliefs towards smoking	s) Smoking cigarettes is expensive	1	2	3	4	5
	t) Smoking shisha is expensive					
	u) Smoking cigarettes makes your teeth look yellow	1	2	3	4	5
	v) Tobacco companies are very bad	1	2	3	4	5
	w) Choosing not to smoke shisha is a cool choice	1	2	3	4	5
	x) Choosing not to smoke cigarettes is a cool choice	1	2	3	4	5

			Yes	No
Q34	I think someone my age who does <u>not</u> smoke cigarettes looks:<br><br>For each statement circle to tell us your view.	a) Normal	1	0
		b) Grown up	1	0
		c) Cool	1	0
		d) Smart (well presented)	1	0
		e) Strong-willed	1	0
		f) Stupid	1	0
		g) Boring	1	0
		*Other*___________________		

EMPOWERMENT

No.	Questions	Categories and coding				
Q35	Please say whether you agree or disagree with each of the statements below. Please don't leave any out. *(Tick all that apply)*	Strongly agree (1)	Agree (2)	Neutr (3)	Disagree (4)	Strongly disagree (5)
	a) I feel able to make choices about what I like and do not like for myself	1	2	3	4	5
	b) I'm confident I can say no to things I do not want to do, even when everyone else is doing it	1	2	3	4	5
	c) I have sources of support outside my family	1	2	3	4	5
	d) My friends support all my decisions, even if they do not agree with them	1	2	3	4	5

SOCIAL NORMS

No.	Questions	Categories and coding				
Q36	Please say whether you agree or disagree with each of the statements below.	Strongly agree (1)	Agree (2)	Neutr (3)	Disagree (4)	Strongly disagree (5)

	a) Most people my age smoke tobacco	1	2	3	4	5
	b) Most people my age smoke shisha	1	2	3	4	5
	c) People my age know the health consequences of smoking tobacco	1	2	3	4	5
	d) People my age ignore most anti-smoking tobacco campaigns	1	2	3	4	5
	e) People my age feel pressure to try smoking cigarettes	1	2	3	4	5
	f) People my age feel pressure to try smoking shisha	1	2	3	4	5
	g) It's easy for someone my age to get cigarettes	1	2	3	4	5
	h) It's easy for someone my age to get shisha	1	2	3	4	5

Q38	Please say whether you agree or disagree with the following statements related to what your friends think:	Strongly agree (1)	Agree (2)	Neutr. (3)	Disagree (4)	Strongly disagree (5)
	a) My friends think I should smoke whenever they are smoking	1	2	3	4	5
	b) My friends think I'm cool if I say no to things I don't want to do even when everyone else is doing it	1	2	3	4	5

Q39	Imagine a close friend was offered a cigarette, and he/she took it. These statements give some reasons why he/she might have done this. Do you agree or disagree with them?	Strongly agree (1)	Agree (2)	Neutr. (3)	Disagree (4)	Strongly disagree (5)
	a) To fit in with a group	1	2	3	4	5
	b) To become more popular with girls	1	2	3	4	5
	c) To become more popular with boys	1	2	3	4	5
	d) To show they can make decisions for themselves	1	2	3	4	5
	e) To be more rebellious towards teachers or parents	1	2	3	4	5
	f) To feel more grown up	1	2	3	4	5
	g) To "stand out" from the group	1	2	3	4	5
	h) Because they can't afford cigarettes themselves	1	2	3	4	5
	i) Because it would be rude to refuse	1	2	3	4	5

Q40	Imagine a close friend was offered shisha, and he/she took it. These statements give some reasons why he/she might have done this. Do you agree or disagree with them?	Strongly agree (1)	Agree (2)	Neutr. (3)	Disagree (4)	Strongly disagree (5)

	a) To fit in with a group	1	2	3	4	5
	b) To become more popular with girls	1	2	3	4	5
	c) To become more popular with boys	1	2	3	4	5
	d) To show they can make decisions for themselves	1	2	3	4	5
	e) To be more rebellious towards teachers or parents	1	2	3	4	5
	f) To feel more grown up	1	2	3	4	5
	g) To "stand out" from the group	1	2	3	4	5
	h) Because they can't afford shisha themselves	1	2	3	4	5
	i) Because it would be rude to refuse	1	2	3	4	5

SOCIAL INFLUENCES

No.	Questions and Filters	Coding	Categories
Q41	In the past two months, have you had a conversation with any adults about smoking?	Yes<br>No	.................1<br>.................0
Q43	In the past two months, have you had a conversation with any of your friends about smoking?	Yes<br>No	.................1<br>.................0
Q44	If Yes to **Q43**: What was the conversation about? *(Tick all that apply)*	They told me about how harmful it is<br>We talked about how I feel about smoking<br>They encouraged me to refuse cigarettes<br>They encouraged me to try smoking	.................1<br>.................2<br>.................3<br>.................4
Q45	Do any of your close family members smoke tobacco?	Yes<br>No	.................1<br>.................0
Q46	If Yes to **Q45**: Which ones? *(Tick all that apply)*	Mother<br>Father<br>Older sister<br>Older brother<br>Someone else in my close family	.................1<br>.................2<br>.................3<br>.................4<br>.................5
Q47	How many of your close friends smoke?	None of my close friends smoke<br>Some of my close friends smoke<br>Most of my close friends smoke	.................0<br>.................1<br>.................2

FOGG MODEL

F1	How easy or difficult would it be for you to say no in case someone asks you to smoke cigarettes?	Very easy<br>Easy<br>Difficult<br>Very difficult<br>DK	……………..1<br>……………..2<br>……………..3<br>……………..4<br>……………..9
F2	How motivated or unmotivated are you to say no in case someone asks you to smoke cigarettes?	Very motivated<br>Motivated<br>Unmotivated<br>Very unmotivated<br>DK	……………..1<br>……………..2<br>……………..3<br>……………..4<br>……………..9
F3	How easy or difficult would it be for you to say no in case someone asks you to smoke shisha?	Very easy<br>Easy<br>Difficult<br>Very difficult<br>DK	……………..1<br>……………..2<br>……………..3<br>……………..4<br>……………..9
F4	How motivated or unmotivated are you to say no in case someone asks you to smoke shisha?	Very motivated<br>Motivated<br>Unmotivated<br>Very unmotivated<br>DK	……………..1<br>……………..2<br>……………..3<br>……………..4<br>……………..9

BEHAVIORS

The next questions ask about your social activities and whether or not you have ever tried tobacco.

No.	Question	Categories			
Q48	How often do you attend:	Never (0)	1-2 times a month (1)	Once a week (2)	More than twice a week (3)
	c. Bars/ Drinking spots	………..0	………..1	………..2	………..3
	d. Restaurants	………..0	………..1	………..2	………..3
	e. Mall	………..0	………..1	………..2	………..3
	f. Funfairs	………..0	………..1	………..2	………..3
	g. Sporting events e.g., interschool competitions, football games etc	………..0	………..1	………..2	………..3
	i. Funerals/ Funeral receptions	………..0	………..1	………..2	………..3
	j. Friends parties or school parties	………..0	………..1	………..2	………..3
	h. Others *(specify)*	………..0	………..1	………..2	………..3
Q49	Have you had alcohol in the last month?	Yes<br>No<br>I've never had alcohol		…………….1<br>…………..0<br>…………….2	

B1	In the last month have you had any of the following?<br>Smirnoff Ice, Beer (Star, Club, etc), Shandy, Brandy, Gin, Bitters, Akpeteshie, Palm wine (fermented), Pito, or any alcoholic drink?	Yes<br>No<br>DK	……………1<br>……………0<br>……………9
Q50	Have you ever tried any of the following?	Yes (1)	No (0)
	a) Cigarettes	1	0
	b) Marijuana/weed	1	0
	c) Snuff	1	0
	d) Hash	1	0
	e) Shisha	1	0
	f) E-Cigarettes	1	0
	g) Khat	1	0
	h) Other tobacco products	1	0
	i) Paper without tobacco	1	0
Q51	How old were you when you first tried smoking (any of the above)?	|___|___|	Don't remember ….99<br>I've never smoked ….0<br>Prefer not to say ……1
Q52	How many times have you tried cigarettes?	Never<br>Just once<br>Two or three times<br>Lots of times	……………….0<br>……………….1<br>……………….2<br>……………….3
Q53	If >0 to Q52, Who gave you the cigarettes when you first tried them? *(Tick all that apply)*	A close friend<br>A relative<br>A boy<br>A girl<br>A teacher<br>A stranger<br>I got it myself<br>Other (specify)…………<br>Prefer not to say	………………1<br>………………2<br>………………3<br>………………4<br>………………5<br>………………6<br>………………7<br>………………8<br>………………9
Q54	If >0 to Q52: In the last month, how many days did you smoke cigarettes?<br>*Circle the correct category.*	0 days<br>1 or 2 days<br>3 to 5 days<br>6 to 9 days<br>10 to 19 days<br>20 to 29 days<br>Every day (all 30 days)	………………1<br>………………2<br>………………3<br>………………4<br>………………5<br>………………6<br>………………7
Q55	In the last three months, has anyone offered you a cigarette?	Yes, someone my age<br>Yes, an adult<br>No	………………1<br>………………2<br>………………0
Q56	If Yes to **Q55**: Did you take it?	Yes<br>No	………………1<br>………………0

Q57	If No to **Q56**: How did you feel about saying no to the offer? (Tick all that apply)	Proud<br>Happy<br>Sad<br>Uncool<br>Other (specify)	....................1<br>....................2<br>....................3<br>....................4<br>....................5
Q58	How many times have you tried shisha?	Never<br>Just once<br>Two or three times<br>Lots of times	....................0<br>....................1<br>....................2<br>....................3
Q59	If >0 to **Q58**, who gave you the shisha when you first tried it? *(Tick all that apply)*	A close friend<br>A relative<br>A boy<br>A girl<br>A teacher<br>A stranger<br>I got it myself<br>Other (specify)..........<br>Prefer not to say	..................1<br>..................2<br>..................3<br>..................4<br>..................5<br>..................6<br>..................7<br>..................8<br>..................9
Q60	In the last month, how many days did you smoke shisha?<br>*Circle the correct category.*	0 days<br>1 or 2 days<br>3 to 5 days<br>6 to 9 days<br>10 to 19 days<br>20 to 29 days<br>Every day (all 30 days)	..................1<br>..................2<br>..................3<br>..................4<br>..................5<br>..................6<br>..................7
Q61	In the last three months, has anyone offered you shisha?	Yes, someone my age<br>Yes, an adult<br>No	..................1<br><br>..................2<br>..................0
Q62	If Yes to **Q61**: Did you take it?	Yes<br>No	..................1<br>..................0
Q63	If No to **Q62**: How did you feel about saying no to the offer?	Proud<br>Happy<br>Sad<br>Uncool	....................1<br>....................2<br>....................3<br>....................4

EXPOSURE (Recall of the SKY Girls campaign)

No.	Question		Categories
E1	a. Have you ever heard about SKY Girls?		Yes....1<br>No....0<br>DK....9
	aa. If **E1a** “No” or “DK”: You may have seen the SKY symbol. Do you recall ever seeing this symbol?		Yes....1<br>No....0<br>DK....9
	b. Have you ever read the SKY magazine?		Yes....1<br>No....0<br>DK....9
	bb. If **E1b** “No” or “DK”: The magazine looks like this. Have you seen this magazine before?	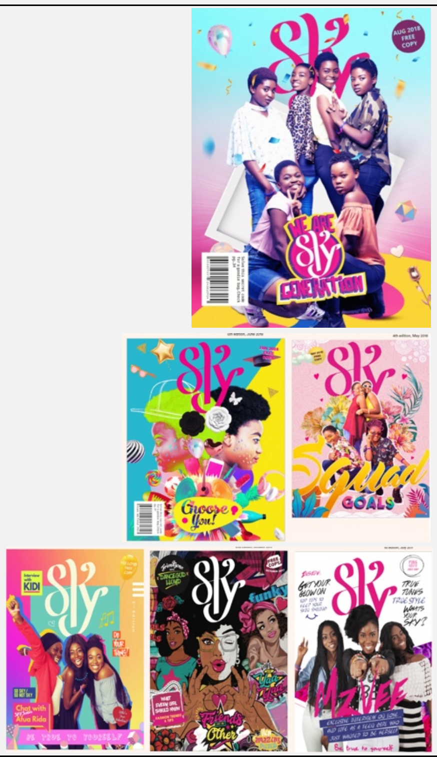	Yes....1<br>No....0<br>DK....9
	c. Has SKY ever visited your school or conducted an activity there?		Yes....1<br>No....0<br>DK....9
	d. Have you watched any of the SKY Movies “Sugar, Spice n’ Sauce”?		Yes....1<br>No....0<br>DK....9

	dd. If **E1d** “No” or “DK”: The movie promo poster looks like this. Do you think you have ever seen the movie?	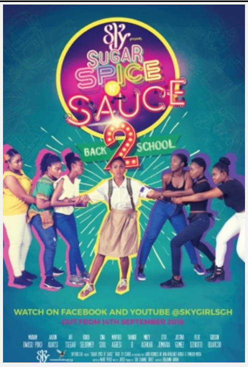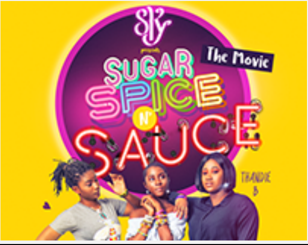	Yes….1<br>No….0<br>DK….9

If “No” or “DK” to all of **E1a, E1aa, E1b, E1bb, E1c, E1d, E1dd**, go directly to **C1**

If at least one “Yes” as a response of any **E1a, E1aa, E1b, E1bb, E1c, E1d, E1dd**, please continue with this section:

EXPOSURE (Recognition of the SKY Girls campaign)

E2	When was the first time that you heard of SKY Girls?	More than a year ago<br>Between a year and 6 months ago<br>Less than 6 months ago<br>DK	……..1<br>……..2<br>……..3<br>……..9
E3	What do you think SKY Girls is about? (*Do not read the options to the participant. Select all the words that are mentioned*)	Girls<br>Empowerment<br>Be/being true to yourself<br>Making positive choices<br>Knowing what's your thing and not your thing<br>Being unique<br>Smoking/not smoking<br>Stay true to yourself<br>Other (specify) ________________<br>DK	……..1<br>……..2<br>……..3<br>……..4<br>……..5<br>……..6<br>……..7<br>……..8<br>……..9
E4	Have you taken the SKY Girls pledge or signed up to become a SKY Girl?	Yes….1<br>No…0<br>DK…9	
E5	If Yes to **E4**: How long ago did you take the pledge?	More than a year ago<br>Between a year and 6 months ago<br>Less than 6 months ago	……..1<br>……..2<br>……..3

		DK	……..9
E6	Are the following statements part of the SKY pledge?<br>a. I pledge to be more competitive in school<br>b. I pledge to be true to myself and what I believe in<br>c. I pledge to respect my parents<br>d. Knowing what's my thing and what's not my thing helps make me who I am	Yes...1 No...0 DK...9<br>Yes...1 No...0 DK...9<br>Yes...1 No...0 DK...9<br>Yes...1 No...0 DK...9	

If at least one "Yes" as a response of any **E1a, E1aa, E1b, E1bb, E1c, E1d, E1dd**, please continue with this section:

RECALL EXPOSURE TO SPECIFIC SKY COMPONENTS

No.	Question	Categories	
E7	If "Yes" to **E1b or E1bb**:<br>You said that you have read the SKY magazine, how many editions have you read?	Only 1….1<br>2-3…..2<br>4 or more….3<br>DK…..9	
E8	If "Yes" to **E1b or E1bb**: What do you remember the most about the magazines? *(open-ended)*		
E9	Have you ever listened to SKY Live on YFM, Facebook, or WhatsApp?	Yes….1<br>No…0<br>DK…9	
E10	If Yes to **E9**, how often do you listen to SKY Live? Would you say you it is every week, 2-3 times a month, once a month or less often?	Never…1<br>Less often…2<br>Once a month…3<br>2-3 times a month…4<br>Every week…5	
E11	Have you ever attended a SKY party at a mall?	Yes….1<br>No…0<br>DK…9	
E12	If Yes to **E11**, how many times?	|___|___|	
E13	If Yes to **E1c**,<br>You said that SKY has visited your school. How many times has SKY visited your school?	Only once…1<br>2 or 3 times…2<br>4 or more times…3	

E14	Which activities has SKY conducted at your school?<br>*APPLY*	SKY Girls introduction in my school<br>SKY Girls movie showing<br>SKY Do Your Thing talent competition<br>SKY Club in my school<br>SKY Magazine drop off or distribution<br>SKY High School Tour with celebrity appearances	Yes.1 No.0 DK.9<br>Yes.1 No.0 DK.9<br>Yes.1 No.0 DK.9<br>Yes.1 No.0 DK.9<br>Yes.1 No.0 DK.9<br>Yes.1 No.0 DK.9
E15	Do you know if there is a SKY Girls Club in your school?	Yes….1<br>No…0<br>DK…9	
E16	Are you a member of a SKY Club in your school?	Yes….1<br>No…0<br>DK…9	
E17	Have you ever watched a SKY vlog episode?	No, never…1<br>Yes, sometimes…2<br>Yes, many times…3	
E18	Do you receive text messages from SKY Girls?	Yes….1<br>No…0<br>DK…9	
E19	If yes, what have the text messages said? *(open-ended*)		
E20	Have you seen a SKY truck in your neighbourhood?	Yes….1<br>No…0<br>DK…9	
E21	Have you heard the SKY song "Unstoppable" by Cina Soul and SKY Girls?	Yes….1<br>No…0<br>DK…9	
E22	If Yes to **E21**, can you repeat any of the lyrics?	Yes….1<br>No…0<br>DK…9	
E23	If Yes to **E22**, what words from the song stand out most for you? (open ended)		
E24	If Yes to **E1d or E1dd**:<br>You said the at you have watched a SKY Movie "Sugar, Spice n' Sauce."<br>Which movie have you seen?	The first one only "Sugar, Spice n' Sauce"…1<br>The second one only "Sugar, Spice n' Sauce: Back 2 school" …2<br>Both….3<br>DK…9	

E25	If Yes to **E1d or E1dd**, who was your favourite character? (do not read out the options)	Maame…1<br>Alice…2<br>Naa…3<br>Marcia…4<br>Mina…5<br>Ama…6<br>Nicole…7<br>Kwamena…8<br>Charles…9<br>Aboagye…10<br>Damien…11<br>Mr Dovlo…12<br>Maame's father…13<br>Maame's mother…14<br>Marcia's mother…15<br>Can't remember…99	
E26	Do you follow SKY Girls on social media?	Yes...1 No...0 DK...9	
E27	If Yes **E26**, in which media?	Facebook<br>Instagram<br>YouTube	Yes.1 No.0 DK.9<br>Yes.1 No.0 DK.9<br>Yes.1 No.0 DK.9
E28	Do you belong to a SKY group on WhatsApp?	Yes...1<br>No...0<br>DK...9	

If at least one "Yes" as a response of any **E1a, E1aa, E1b, E1bb, E1c, E1d, E1dd**, please continue with this section:

RECOGNITION OF SKY MESSAGES

	I am going to read you some statements about SKY Girls and I would like for you to tell me if you "strongly agree", "agree", are "neutral," "disagree" or "strongly disagree" with each of them.		
R1	SKY girls is about making decisions that you feel are right for you	SD…1 D…2 N…3 A…4 SA…5 DK..9	
R2	SKY Girls is about being true to yourself	SD…1 D…2 N…3 A…4 SA…5 DK..9	
R3	SKY Girls is about being good at sports	SD…1 D…2 N…3 A…4 SA…5 DK..9	

R4	SKY Girls is about doing your thing	SD…1 D…2 N…3 A…4 SA…5 DK..9	
R5	SKY Girls is about spending time on your own	SD…1 D…2 N…3 A…4 SA…5 DK..9	
R6	Dancing is a popular SKY Girls thing	SD…1 D…2 N…3 A…4 SA…5 DK..9	
R7	Smoking is not a SKY Girls thing	SD…1 D…2 N…3 A…4 SA…5 DK..9	
R8	SKY generation is 100% swagged up/no shisha	SD…1 D…2 N…3 A…4 SA…5 DK..9	

If at least one "Yes" as a response of any **E1a, E1aa, E1b, E1bb, E1c, E1d, E1dd**, please continue with this section:

SATISFACTION WITH SKY

S1	How often do you talk with your friends about SKY or any SKY content you have seen?	Very often...4<br>Often...3<br>Sometimes...2<br>Rarely...1<br>Never...0	
S2	Who do you talk about SKY with?<br>(Select all that apply)	My close girl friends only…1<br>Girls outside my close group of friends…2<br>Boys…3<br>With family members…4	
S3	On a scale of one to five, where 1 is 'not at all important' and 5 is 'very important', how important is SKY Girls to you?	Not at all important…1<br>Not important…2<br>Indifferent…3<br>Important…4<br>Very important…5	
S4	Of the different ways of connecting with SKY Girls, which is the most important to you?	SKY Live …1<br>SKY Magazine…2<br>SKY Girls on Facebook…3<br>SKY Girls on Instagram…4<br>SKY Girls via WhatsApp…5<br>SKY Girls Takeover parties at malls…6<br>SKY Girls at my school…7<br>SKY Girls on YouTube…8	

	Did SKY help you to do any of the following things? Please reply with yes or no.		
S5	a. Feel confident to make decisions for myself	Yes...1 No...0 DK...9	
S6	b. Connect with friends and other girls my age	Yes...1 No...0 DK...9	
S7	c. Think about my future and what I want	Yes...1 No...0 DK...9	
S8	d. Be inspired by other girls and women	Yes...1 No...0 DK...9	
S9	e. Reject shisha or cigarettes if offered by someone	Yes...1 No...0 DK...9	

All participants should respond to this section:

CONFOUNDING PROGRAMS

No.	Question	Categories	
C1	Do you know of any organizations, programs, or club that encourage young girls to express themselves?	Yes...1<br>No...0<br>DK...9	
C2	If Yes to **C1**, how did you hear about it or about them?	Social media...1<br>School....2<br>Church....3<br>Government organization....4<br>NGO...5<br>Radio...6<br>TV...7<br>Newspaper or magazine...8<br>Poster/street add/Flyer...10<br>Others...11<br>DK...9	
C3	If Yes to **C1**, do you know if these organizations, programs, or clubs address smoking? *(ask for each club participant mentioned)*	Yes...1<br>No...0<br>DK...9	

C4	If Yes to **C1**, have you participated in any of them?	Yes...1<br>No...0<br>DK...9	
C5	If Yes to **C4**, which ones? (open-ended)		
C6	If Yes to **C4**, how many of the organizations, programs, or clubs that you participated in addressed smoking?	None...0<br>One...1<br>More than one...2	
